# Medical laboratory diagnostics in Germany – a status report 2024

**DOI:** 10.3205/000337

**Published:** 2025-04-09

**Authors:** Michael Vogeser, Timo Schumacher, Frank Bühling

**Affiliations:** 1 Institute of Laboratory Medicine, LMU University Hospital, LMU Munich, Munich, Germany; 2 Practice Schumacher, Langer, Schumacher, Schwanewede, Academic Teaching Practice of the University of Göttingen and Hannover Medical School, Schwanewede, Germany; 3 labopart – Medical Laboratories, Dresden, Germany

**Keywords:** in vitro diagnostics (IVD), laboratory diagnostics, laboratory medicine, diagnostics, health services research, Germany, statistics

## Abstract

**Background and aim of the work:**

Laboratory diagnostics (in-vitro diagnostics, IVD) is one of the main pillars of evidence-based medicine; for many medical fields – such as endocrinology – laboratory tests are conditional, but not indispensable for any discipline. The majority of diagnoses can only be reliably made if laboratory tests are taken into account. The aim of this study is to describe the provision of IVDs to the population in Germany in an overall view and to discuss development perspectives in the sense of basic healthcare research, particularly on the basis of publicly accessible data.

**Methods:**

For this purpose, in particular the federal health reporting, reports of the German Federal Statistical Office, the medical register of the German Medical Association and data from the Associations of Statutory Health Insurance Physicians were analysed, as well as a large amount of publicly accessible information from associations and medical institutions.

**Results:**

The provision of laboratory services in Germany is strongly interlinked between the sectors as they are offered by practicing laboratory physicians, in inpatient facilities and also directly in the practices and medical care centres of other specialist groups. There are currently around 1,200 specialists in laboratory medicine and 840 specialists in microbiology, which together equates to around 0,7% of all specialized physicians in Germany. Around 2/3 of laboratory physicians work in private practice. After the group of general practitioners, laboratory doctors are the second most frequently consulted group of doctors in Germany as representatives of a non-curative specialty. A total of around 108,000 people work in medical laboratories (1.8% of the entire healthcare workforce). The annual cost of laboratory diagnostics is around 150 euros per capita, totaling around 12.9 billion euros per year, which corresponds to around 2.6% of the total costs of the German healthcare system. Only around 17% of hospitals – predominantly maximum care facilities – have their own laboratory infrastructure, while the majority of hospitals are supplied with laboratory services by practicing laboratory doctors. In the latter, it is now predominantly quite large laboratory medicine units that provide laboratory tests nationwide on the basis of a complex logistics and data infrastructure, which is flanked by patient-related laboratory diagnostics in practices and medical care centres in a wide range of disciplines.

**Conclusion:**

A fairly comprehensive picture of the provision of laboratory services in Germany can be obtained from publicly accessible sources. Laboratory diagnostics is an essential and efficient, system-relevant element of the German healthcare system, in which a relatively small number of medical specialists and healthcare professionals bear a great degree of responsibility for maintaining adequate care.

## Zusammenfassung

**Hintergrund und Ziel der Arbeit:**

Labordiagnostik (In-vitro-Diagnostik, IVD) ist eine der wesentlichen Säulen der evidenzbasierten medizinischen Versorgung; für viele medizinische Bereiche – wie beispielsweise die Endokrinologie – sind Laboruntersuchungen konditional, jedoch für kein Fach verzichtbar. Die Mehrzahl der Diagnosen kann nur unter Berücksichtigung von Laboruntersuchungen zuverlässig gestellt werden. Ziel der vorliegenden Arbeit ist es, im Sinne grundlegender Versorgungsforschung insbesondere auf Basis öffentlich zugänglicher Daten die Versorgung der Bevölkerung in Deutschland mit IVD in einer Gesamtschau zu beschreiben und Entwicklungsperspektiven zu diskutieren.

**Methoden:**

Hierfür wurden insbesondere die Gesundheitsberichterstattung des Bundes und des Statistischen Bundesamtes, das Arztregister der Bundesärztekammer sowie Daten der Kassenärztlichen Vereinigungen ausgewertet, zudem eine große Zahl von öffentlich zugänglichen Informationen von Verbänden und medizinischen Institutionen.

**Ergebnisse:**

Die Versorgung mit Laborleistungen erfolgt in Deutschland stark zwischen den Sektoren verzahnt durch niedergelassene Laborärzte, in stationären Einrichtungen sowie auch direkt in den Praxen und medizinischen Versorgungszentren (MVZs) anderer Facharztgruppen. Derzeit sind ca. 1.200 Fachärztinnen und Fachärzte für Labormedizin und 840 Fachärztinnen und Fachärzte für Mikrobiologie tätig, was zusammen ca. 0,7% aller Fachärzte entspricht. Etwa 2/3 der Laborärztinnen und Laborärzte sind im niedergelassenen Bereich tätig. Nach der Gruppe der Allgemeinmediziner sind die Laborärztinnen und Laborärzte – als Vertreter eines nicht-kurativen Fachs – die am zweithäufigsten konsultierte Arztgruppe in Deutschland. Insgesamt arbeiten etwa 108.000 Personen in medizinischen Laboratorien (1,8% des gesamten Gesundheitspersonals). Die jährlichen Ausgaben für Labordiagnostik liegen bei etwa 150 Euro pro Kopf, in der Summe bei ca. 12,9 Mrd. Euro jährlich, was ca. 2,6% der gesamten Kosten des deutschen Gesundheitswesens entspricht. Noch ca. 17% der Kliniken – überwiegend Maximalversorger – verfügen über eine eigene Laborinfrastruktur, während die Mehrzahl der Kliniken aus dem niedergelassenen laborärztlichen Bereich mit Laborleistungen versorgt wird. In diesem Bereich realisieren mittlerweile überwiegend recht große labormedizinische Einheiten auf Basis einer komplexen Logistik- und Dateninfrastruktur die flächendeckende Versorgung mit Laboruntersuchungen, die von patientennaher Labordiagnostik in Praxen und Medizinischen Versorgungszentren unterschiedlicher Disziplinen flankiert wird.

**Schlussfolgerung:**

Aus öffentlich zugänglichen Quellen kann ein recht umfassendes Bild der Versorgung mit Laborleistungen in Deutschland gewonnen werden. Die Labordiagnostik stellt ein wesentliches und effizientes, systemrelevantes Element des deutschen Gesundheitswesens dar, bei dem eine relativ gesehen kleine Anzahl von Fachärztinnen und Fachärzten sowie medizinischen Fachkräften eine große Verantwortung für die Aufrechterhaltung einer angemessenen Versorgung trägt.

## Introduction

The aim of this publication is to provide the medical public with a comprehensive overview of the structures and processes of medical laboratory diagnostics in Germany – as a fundamental contribution to health services research in the field of laboratory diagnostics. The work is a follow-up to a previous survey [1].

The results of laboratory tests are important for a very large proportion of medical decisions of various kinds – although quantitative statements on this cannot be made reliably. Laboratory tests are relevant with regard to making a diagnosis in a treatment situation – usually as a component of a “mosaic diagnosis” – but also with regard to early detection before a disease manifests, the detection of disease predispositions, prognostics and therapy monitoring. The importance of laboratory diagnostics often relates to the dominant causes of treatment, sometimes also to environmental aspects and secondary diagnoses. In most individual clinical pathways, reliable and readily available laboratory results are a key determinant of patient safety.

## 1 Basic discipline structures and working methods

Human sample material that is analyzed at a distance (more or less) from the patient is the subject of in-vitro diagnostics (IVD). The range of materials examined is very broad (blood, urine, cerebrospinal fluid, stool, punctates, secretions, biopsies, surgical specimens, smears, ejaculate, hair, saliva, concretions, and more).

The specialist field of laboratory medicine is dedicated to IVD in the broadest sense. The specialist field of microbiology focuses on the diagnosis of pathogen-related diseases. The specialist field of pathology particularly addresses diagnostic tissue samples (surgical specimens, biopsies), as well as macroscopic-anatomical diagnostics, i.e. the performance of autopsies for the purposes of medical quality assurance and cell-related examinations (cytology). What these disciplines (laboratory medicine, microbiology, pathology) have in common is that there is no direct patient contact and no immediate treatment (non-curative, secondary medicine). The focus is on contact with the attending physicians, who send the respective samples to laboratories with diagnostic questions. In the specialist field of transfusion medicine, the focus is on the production and provision of blood products and cell therapeutics, based on the use of IVDs. In the specialist field of human genetics – in addition to IVD – clinical diagnostics and counseling of patients and those seeking advice play an important role. 

Primary curative disciplines in which – depending on the institution – complex IVD is carried out quite extensively are, in particular, haematology and clinical toxicology (as sub-areas of internal medicine). Endocrinology (as a branch of internal medicine), gynecology and urology should also be mentioned. The performance of IVD is addressed here by the respective further training regulations, whereby additional qualifications must be demonstrated for authorization for the respective IVD. General laboratory tests of limited complexity are also carried out to a greater extent by general practitioners, paediatricians, internists (general practitioners, but also semi-specialists such as rheumatologists and immunologists), dermatologists and ENT specialists (especially in the field of allergy diagnostics). This takes place partly in the doctor’s own practice (practice laboratory), but also partly in functionally outsourced, jointly operated practice laboratories as so-called “Laborgemeinschaften” (shared laboratories). In particular, the indication and diagnosis are carried out here by the doctors of the specialist disciplines mentioned. Depending on the institution – particularly in the university sector – IVD also plays an important role in occupational and environmental medicine, as well as in clinical pharmacology. This also applies in part to forensic medicine with forensic toxicology, although this is not primarily curative in nature.

In the complex landscape of specialist areas (and additional qualifications), the specialist area of laboratory medicine has a special role, as this area is generalist in terms of IVD in that it can also address services that are provided by specialists in microbiology, for example. A not inconsiderable number of doctors have two specialist titles in IVD, primarily laboratory medicine and microbiology.

Medical laboratory diagnostics has a very high degree of automation overall. The core elements of medical laboratories are fully automated analyzer systems, typically separate for the underlying examination techniques of photometry, immunoassay, cell counting and coagulometry. These systems are used to process large numbers of standard analyses with relatively low staffing levels. In addition, smaller systems are used for less frequently requested tests; semi-automated or purely manual procedures are also used, such as various microscopic analyses or complex procedures such as chromatographic analyses to determine drug levels. 

In larger laboratories, comprehensive automation systems are often used, which implement closed sample processing from sample registration to pre-analysis with centrifugation and aliquoting, actual analysis and sample archiving. The creation of findings is largely IT-supported and includes technical and medical validation, including possible text-based reporting for more complex procedures by medical staff. Highly automated systems are now also available in microbiology and transfusion medicine. Key areas of responsibility of medical laboratories in outpatient care also include transport logistics, usually through their own transport services, as well as data links with senders. In particular, laboratory work includes advising senders on diagnostic procedures, strategies and interpretation of findings, individual reporting of special analyses, ongoing quality assurance, training and supervision of staff and overall monitoring and structuring of complex interlinked process chains – with ultimate responsibility for valid, value-adding laboratory findings.

## 2 Service providers in laboratory diagnostics

### 2.1 Physicians

According to the medical register of the German Medical Association [2], there were 1,206 doctors of laboratory medicine and 845 doctors of microbiology working in Germany at the end of 2023 (Table 1 (Tab. 1)). Together, this corresponds to approx. 0.7% of all working specialists and 0.5% of all working doctors. Compared to 2010, the number has increased by approx. 23%, compared to an increase in the number of all doctors of approx. 28% in this period. The proportion of women in laboratory medicine and microbiology is approx. 44%, compared to approx. 50% in the overall group of specialists. Around 33% of these specialists are over 60 years old, compared to a corresponding proportion of 23% in the entire German specialist medical profession.

According to the National Association of Statutory Health Insurance Physicians (Kassenärztliche Bundesvereinigung, KBV), 153,726 doctors were involved in statutory health insurance (SHI) accredited medical care (vertragsärztliche Versorgung) on 31/12/2023 [3]. This included 1,378 physicians in the specialist group “Laboratory medicine/biochemistry/microbiology”; 120 with a license as a panel doctor (Vertragsarzt), 1,118 employed in medical care centres (medizinische Versorgungszentren, MVZs), 51 employed in private practice. 77 doctors had additional specialist training in laboratory diagnostics.

According to the KBV Quality Report 2023 [4], 10,426 doctors from different specialties had a license to carry out examinations in the area of special laboratories (e.g. gynaecologists to carry out hormone analyses), in accordance with the Special Laboratory Quality Assurance Agreement [5] pursuant to Section 135 (2) of the German Social Code (SGB) V.

The number of doctors undergoing further training in laboratory diagnostics is not known. There are also no figures on non-physician academics working in medical laboratories.

### 2.2 Non-scientific personnel

According to the Federal Statistical Office ([6], search term: health workforce calculation, 23621, 23621-0003 (health workforce: Germany, years, employment, age groups, healthcare professions; available period 2012–2022); 23621-0008 (health workforce (full-time equivalents): Germany, years, age groups, healthcare occupations), a total of 77,000 full-time equivalents were recorded in medical laboratories in 2022, of which 50,000 were qualified staff (71%), 2,000 full-time equivalents are listed as specialists. In addition to the professional groups of medical technical assistants (Medizinisch-technische Assistenten, MTA)/medical technologists for laboratory analysis (Medizinische Technologen für Laboratoriumsanalytik, MTL), medical laboratories also employ workers without a specific laboratory diagnostic qualification, e.g. in the secretarial area, in transport logistics or for activities in sample reception. Thus, approx. 2% of the full-time equivalents of all healthcare personnel in Germany were employed in medical laboratories.

Compared to 2012, the total full-time equivalent number has increased by 4%, while the number of qualified personnel has decreased by 6%. 

The total number of personnel employed in medical laboratories in 2012 was 98,000 (2% of the total healthcare workforce), including 69,000 qualified staff; in 2022, the total number was 108,000 (1.8% of the total healthcare workforce), including 72,000 specialists. The proportion of professionals employed full-time in medical laboratories fell from 55% to 47% between 2012 and 2022, from 38,000 to 34,000 people in absolute terms [6].

In hospitals, 21,000 professionals were employed in medical laboratories in 2012; in 2022, the number was 15,000 (a decrease of 29%) [6].

The Medical Technology Professions Act (MT-Berufe-Gesetz – MTBG) regulates who is authorized to carry out laboratory tests as part of the medical profession. These are primarily recognized medical technologists for laboratory analysis (MTL) in relation to the so-called reserved activities (§ 5), i.e. the performance of biomedical analysis processes using biological, chemical and physical methods and procedures, including plausibility checks, validation and quality assurance. The reserved activities do not include simple quantitative and qualitative laboratory analyses.

Exceptions with regard to the reserved activities apply to persons who have the necessary specialist knowledge, skills and abilities to carry out the aforementioned activities on the basis of a completed university education, as well as non-medical practitioners. People with Bachelor’s degrees in biotechnological disciplines are becoming increasingly important. Persons who have completed other medical training and who work under the supervision and responsibility of a person named in the previous sentence may also be authorized to carry out reserved activities. For example, a medical assistant or a healthcare assistant may carry out laboratory tests under the supervision and responsibility of a doctor. A food chemist, for example, is authorized to carry out chromatographic tests in a special laboratory, as this type of analysis is addressed in the degree course. For the physician, laboratory services and supporting measures such as blood sampling can generally be delegated (which is particularly relevant in the case of point-of-care testing (POCT)). However, the indication for examinations, the diagnosis, as well as the explanation and counseling of patients, including taking a medical history, are not delegable services of the physician – including in laboratory diagnostics.

The physician’s privilege for the preparation of valid medical laboratory reports is only found in a few countries apart from Germany.

During routine working hours, in the presence and under the supervision of medical staff, medical assistants are increasingly working in medical laboratories – in accordance with the above-mentioned requirements – while in clinics only MTLs are allowed to work at night – without the presence of a responsible physician. There is increasing competition between laboratory practices and hospital laboratories for MTLs – as a shortage occupation. In hospital laboratories, the workload is typically higher due to shift work and night shifts, although pay tends to be better than in the outpatient laboratory diagnostics sector.

## 3 Sectors of care

### 3.1 Clinics

The Statistical Report Basic Data on Hospitals 2022 ([7], Table 23111-30), shows a total of 1,893 hospitals, of which 1,128 have at least one non-bed-carrying specialist department. There was a specialist department for laboratory medicine in 331 hospitals (17%) and a specialist department for transfusion medicine in 91. Specialist departments for pathology were recorded in 147 hospitals, as well as 714 specialist departments for radiology. The statistics do not show how many of the specialist departments for laboratory medicine are staffed by specialists in laboratory medicine.

Among the 613 hospitals with fewer than 300 beds, 15% had a specialist department for laboratory medicine, 36% of hospitals with between 300 and 599 beds, and 117 specialist departments were listed for hospitals with more than 600 beds, which corresponds to a share of 67%. Since the operation of such large hospitals is inconceivable without extensive laboratory care, these data demonstrate that in many large hospitals the respective laboratory is operated externally and not under the hospital’s own responsibility, and thus cross-sectoral forms of care exist, i.e. the supply of hospital operations by laboratories in the private practice sector, supplemented by procedures for patient-oriented immediate diagnostics (POCT).

The Federal Joint Committee’s (Gemeinsamer Bundesausschuss, G-BA) quality structure guidelines require the constant availability of laboratory physicians for a larger number of services in hospitals, including for perinatal centres of higher categories, as well as with regard to the billing of the intensive care lump sum. 

### 3.2 Outpatient sector

Outpatient laboratory diagnostic care is mainly provided by specialist outpatient laboratories. As a rule, several colleagues work in these laboratory practices (often 5–10), frequently laboratory physicians and microbiologists together, sometimes also pathologists, geneticists, transfusion physicians, often constituted as medical care centres (MVZ). To a certain, usually small extent, these practices have patient traffic for sample collection – assigned, among others, by specialists who do not maintain blood collection logistics. In addition to so-called owner-managed medical laboratories, the majority of laboratory practices and MVZs are now networked in various legal forms, regionally, nationally and internationally. In the laboratories of commercial laboratory providers, the medical staff are employed in the same way as in hospitals. Typical laboratory MVZs today supply several hundred doctors’ practices and often also numerous clinics. Accordingly, sample transportation logistics are of central importance. The transportation service is either a separate functional unit of these practices or is operated by external service providers.

In addition to specialist laboratory practices, there are also so-called laboratory cooperatives (Laborgemeinschaften) – as outsourced practice laboratories run jointly by several doctors and operated by MTL. Corresponding constructs – in which the partners of the laboratory consortium bear the medical responsibility for the performance and reporting of basic parameters – are often functionally integrated into specialist laboratories, including transport logistics.

According to a 2020 publication by the consulting firm Aktiva [8], the share of commercial laboratory supplier chains was 55% of total sales, which is reported at € 10.7 billion. Of this, Sonic Healthcare/Bioscientia accounted for 14%, Synlab for 12%, the Limbach Group for 11% and amedes for 8%. The largest global laboratory diagnostics groups are not yet active in Germany (in particular Quest Diagnostics and Laboratory Corporation of America (Labcorp)).

Laboratory physicians are subject to demand planning [9] with regard to participation in statutory health insurance provision. The subject is assigned to “separate specialist care”, in which the area of the respective Association of Statutory Health Insurance Physicians is regarded as the planning area (i.e. essentially the federal states). In all planning areas, a supply of over 100% is currently determined, i.e. there is a de-facto establishment block [10]. The (annually recalculated) demand planning is based on the G-BA’s demand planning guideline (separate specialist care), which lists the ratios between residents and specialists in a particular discipline, taking morbidity into account, among other things. This shows that, on average, one laboratory doctor is required to provide laboratory diagnostic care for 92,218 people [11].

Many doctors’ offices in a wide range of specialist disciplines operate practice laboratories in which – in addition to blood sampling and pre-analytical preparation of samples – various laboratory tests are carried out, some of which cover a wide spectrum (POCT, point-of-care testing). Typical procedures are glucose measurement, blood sedimentation, urine strip test; in addition, a growing range of quantitative single-sample measurement procedures, e.g. for infarct markers (troponin), D-dimer, BNP, CRP, and Marcumar monitoring. There are also an increasing number of systems available for more specialized tests, such as the measurement of HbA1c, which are intended for use in practice laboratories. In MVZs with several disciplines, automated multi-channel analysis systems are also used in some cases, including in the field of endocrinology and reproductive medicine. This means that the practice laboratory accounts for a fairly large proportion of laboratory care in Germany. This area is considered to be growing, although no objective data is available. With regard to tests commissioned from laboratory colleagues, a specialist practice generally works with just one laboratory practice. These ensure the transport logistics (including the provision of collection materials) and the electronic feedback of reports. The interfaces for remote data transmission to the practice software systems are an important element here.

## 4 Volume of services

Direct data on the total number of laboratory tests performed annually in Germany is not publicly available. According to the BDL (Berufsverband Deutscher Laborärzte e.V.), around 9 million laboratory results are produced every day in Germany.

For the first quarter of 2023 [12], the Central Institute of Statutory Health Insurance Physicians (Zentralinstitut kassensärztliche Versorgung) states a total of 577,634,988 treatment cases, including 76,995,362 cases for laboratory physicians, although the number of analyses per case is not specified.

For 2022, the Scientific Institute of the AOK [13] states the number of billed services from the uniform scale of assessment (Einheitlicher Bewertungsmaßstab, EBM) laboratory section at 1.49 billion (Chapter 32.01, basic services 439 million; Chapter 32.02 838 million; Chapter 32.03, special examinations, 211 million) as an evaluation of the SHI frequency statistics. A figure of 1.58 billion is given for 2017 [14]. These figures only relate to outpatient services for people with statutory health insurance; no figures are available for the inpatient sector.

In this evaluation by the Scientific Institute of the AOK [13], the 20 most frequent billing items are also specified for the different groups of doctors. For the field of laboratory medicine, these 20 items accounted for a cumulative 73% of all examinations in 2022. These include 22.4 million analyses of mechanized blood counts (item 1), 15.8 million TSH determinations (item 6), and 12.3 million HbA1c measurements (item 12). The report also provides an indication of the extent to which specialist disciplines outside of laboratory medicine provide laboratory diagnostic services. For example, nuclear medicine specialists carried out 517,000 TSH measurements and 129,000 vitamin D measurements.

The Barmer Arztreport 2023 [15] lists, among other things, the percentage of affected outpatient billing cases by specialist discipline. According to this, 50.7% of insured persons were concerned by laboratory medicine services in 2022. An increase of 5.8% was observed compared to 2021. The average cost per person here was 35 euros per year.

Tests that can be ordered and paid for by non-laboratory physicians in laboratory cooperatives – as spatially outsourced practice laboratories – include around 40 parameters that can be described as extended basic diagnostics – including HbA1c, TSH and immunoglobulins, for example. This readily available routine spectrum forms a very powerful basis for the curative-diagnostic work-up of patients in Germany. 

The following services are covered by the statutory health insurance companies as preventive check-ups: women and men over 35 every 3 years: total cholesterol, LDL cholesterol, HDL cholesterol, triglycerides, serum glucose; urine strip test: protein, glucose, erythrocytes, leukocytes, nitrite as well as a one-off screening for hepatis B and C. Further preventive examinations are specified in the G-BA guideline on conception regulation and in the Maternity Protection Act. In addition, various health insurance companies have concluded framework agreements on special preventive services, particularly in the context of the care of pregnant women. One example is the “Hallo Baby” framework agreement for special care in accordance with Section 140a SGB V to prevent premature births and infection-related birth complications.

## 5 Costs of laboratory diagnostics

The Federal Statistical Office’s calculation of healthcare costs [16] indicates that expenditure on laboratory services will total € 12.9 billion in 2022, accounting for 2.6% of total healthcare expenditure (Table 2 (Tab. 2)). Per capita expenditure on laboratory diagnostics is therefore around € 150 per year. Expenditure on laboratory services is seen to increase by € 4.5 billion between 2012 and 2022. This corresponds to an increase of 55%, compared to an increase of 63% of all healthcare expenditure in this 10-year period. The overall inflation-related price increase in Germany for this period was around 15%. 

In 2022, statutory health insurance accounted for 65% of expenditure on laboratory services, while private health insurance accounted for 19%.

The Federal Statistical Office publishes comprehensive quality reports on health expenditure accounting and health personnel accounting [17], [18]. The legal basis for data collection is Regulation (EC) No. 1338/2008 on Community statistics on public health and health protection at work.

## 6 Compensation for laboratory services

Clinics – In clinics, laboratory operations are not financed on the basis of individual tests. The clinics essentially receive lump sum payments for patients treated on the basis of the respective diagnosis profile within the DRG system (Disease-Related Groups). This results in a total financial volume from which all functional units of a clinic – including the laboratory – are to be operated.

The Institute for the Hospital Remuneration System (InEK; https://www.g-drg.de) defines which medical services are included in the calculation of the DRG. Laboratory services are taken into account as part of the total costs for treatment, but are not broken down as a separate share. The distribution of revenues generated by DRG per-case flat rates takes place internally in the hospital and depends on the respective negotiations, budgets and structures of the hospital.

Internal cost accounting is established to very different extents in hospitals. In principle, the total expenditure of a hospital laboratory can be determined on the basis of a relative key for the various examination procedures (generally on the basis of the point rules of the fee schedule for doctors, GOÄ) to determine the extent to which individual departments of a hospital generate laboratory costs – in the sense of internal cost allocation. Typically, the proportion of a clinic’s expenditure for laboratory tests is around 2%.

Patients who are treated in outpatient clinics and polyclinics at university hospitals are generally also reimbursed by the clinic on a lump sum basis for patients with statutory health insurance (polyclinic lump sums). 

Laboratory physicians in private outpatient laboratories (who may be employed) bill their services to the statutory health insurance funds in accordance with the EBM (uniform scale of assessment); privately insured patients are billed on the basis of the GOÄ. In both fee scales, the individual services are assigned dedicated billing amounts [19], [20]; in the case of the GOÄ, not all services are currently listed, which is why so-called analog numbers must be used in some cases. The EBM is drawn up within the framework of medical self-administration, while the GOÄ is a state fee schedule.

The Associations of Statutory Health Insurance Physicians are organized at federal state level and are responsible for ensuring the statutory health insurance community’s solidarity (approx. 87% of the population [21], 74.3 million insured persons). The respective associations of statutory health insurance physicians distribute the funds from the statutory health insurance funds to the panel doctors as fees according to a complex system. 

The EBM, which is relevant for those with statutory health insurance, is decided by the evaluation committee and updated on an ongoing basis, including with regard to new services (https://institut-ba.de). The evaluation committee consists of three members appointed by the KBV and three members appointed by the National Association of Statutory Health Insurance Funds (GKV). 

The distribution of total remuneration for statutory health insurance (SHI) is regulated by the German Social Code, Fifth Book (SGB V). In principle, a distinction is made between the morbidity-related total remuneration (MGV) and the remuneration for extra-budgetary services. The MGV part of the remuneration paid by the health insurance funds is divided into different care areas and basic amounts. The distribution is regulated by the fee distribution scales (HVM) of the individual associations of statutory health insurance physicians in consultation with the associations of health insurance funds.

The most important areas of care are: general practitioner care area, specialist care area, basic laboratory fee. In addition, there are further fee volumes for the remuneration of services in emergencies and emergency services, services in the care area of pediatrics and adolescent medicine, the remuneration of psychotherapeutic services and structural contracts.

The efficiency bonus (see below) and the laboratory services ordered are reimbursed from the basic laboratory fee. These are the services referred to the specialist laboratory on the sample 10 form. 

The basic laboratory lump sums are reimbursed from the fee volume of the specialist care area – the actual laboratory fee per treatment case (codes 12220 and 01700); all other codes are used to reimburse specified technical services of a treatment case. 

Self-provided laboratory services and joint laboratory services (i.e. referrals on model 10c) are remunerated from the basic amount for general practitioners and specialists in accordance with the assignment of the doctor providing the service to the respective care area. Laboratory services in the organized emergency service are allocated to the fee volume for the medical on-call service.

All preventive laboratory services (e.g. maternity protection, check-ups, tumour screening, transplant aftercare) and particularly specialized laboratory services are reimbursed from the remuneration for extrabudgetary services. In some cases, new types of laboratory services are also remunerated on an extra-budgetary basis for a certain period and then transferred to the MGV. Services from the extrabudgetary remuneration area are not subject to quotas. 

For processing an examination order, the laboratory doctor initially receives a basic laboratory lump fee (EBM number 32001), irrespective of the scope of the respective requirement profile, which is then covered by further EBM numbers, as well as other lump sums (e.g. transport). The full basic lump sum is paid per specialist for the first 6,000 cases per quarter (code 12220 with 14 EBM points); 4 points are charged for the next 6,000 cases and 1 point for all further cases (“Abstaffelung”). The basic lump sums are subject to an inflation adjustment regulated by the annually adjusted point values. Fixed prices were determined for the remuneration of technical services, which are not subject to regular inflation adjustment.

In addition, the remuneration of all laboratory services may be subject to quotas if the volume of the basic laboratory fee set for the respective KV area is insufficient. A lower limit of 89% is currently set here. The quotas for the basic flat rates and the efficiency bonus are regulated differently in the fee distribution scales of the individual KVs.

At the end of 2023, the Evaluation Committee determined that new flat rates will be included in the EBM from the beginning of 2025, with which the transport of samples, the free provision of collection material and the technology for electronic order placement will be specifically remunerated. To finance this, the remuneration of laboratory services will be reduced as a percentage; this laboratory remuneration reform – which was determined by the self-administration of the healthcare system and not by government regulations – has been heavily criticized by practicing laboratory physicians, as they expect to be financially worse off overall from 2025.

The quarterly KBV fee report of the National Association of Statutory Health Insurance Physicians – which discloses average values for the personal income of physicians in the various disciplines – does not include laboratory physicians (as one of only a few groups of physicians).

From the perspective of practicing specialists outside of laboratory medicine, the principle of remuneration for laboratory services – taking into account the so-called efficiency bonus/laboratory bonus – can be described as follows: for a general practitioner, the following applies: he receives a laboratory bonus of € 2.19 per quarter and per patient (€ 2,190 for 1,000 patients). If he spends <€ 1.60 per patient (in total) on laboratory services in a quarter, he receives this € 2.19 efficiency bonus in full. If he spends more than € 3.80 on laboratories, he no longer receives a bonus. If he spends between € 1.60 and € 3.80, the laboratory bonus paid out will always be lower. If the total laboratory costs in the quarter are >€ 3.80 per patient (i.e. over € 3,800 for 1,000 patients), the laboratory services are remunerated on a case-by-case basis according to EBM, e.g. for a POCT measurement. Approximately 50% of general practitioners are paid more than € 3.80, i.e. they do not receive a laboratory bonus. All services count as laboratory services with regard to the laboratory bonus: those provided in the practice, those provided in a laboratory group and those provided by a laboratory specialist.

Laboratory services that are characterized by a so-called exemption number are excluded from the laboratory budget with regard to the laboratory bonus. In the diagnosis of diabetes, for example, this applies to HbA1c, creatinine and the microalbuminuria test. For other specialist groups, other limits apply for the upper limit with regard to the laboratory bonus, for example significantly higher limits for endocrinologists and gynecologists. If the limits per quarter are exceeded and a laboratory bonus is no longer paid out, the amount of the laboratory costs for the respective doctor is no longer relevant and no sanctions are imposed. However, all costs are generally allocated to all doctors in an association of statutory health insurance physicians, which is subject to budgeting in the distribution of fees. The annual volume of the efficiency bonus – which is not spent on the provision of laboratory services but as a control element – is around 450 million euros.

## 7 Quality assurance and quality reporting

A central role in the quality assurance of laboratory tests in Germany is played by the guidelines of the German Medical Association for the quality assurance of laboratory medical tests, usually referred to as Rili-BÄK in everyday laboratory practice. As a chamber guideline, it is to be regarded as binding under professional law. 

The Medical Devices Operator Ordinance is issued by the Federal Ministry of Health and specifies the obligations for users of diagnostics; in Section 9, it requires the establishment of a quality assurance system for the use of in-vitro diagnostics as a medical device and for the performance of all laboratory tests in medicine; this is considered to be the case if the Rili-BÄK is complied with. The Rili-BÄK also specifies requirements for the organizers of proficiency tests as an element of external quality assurance. The respective supreme state authorities of the federal states are responsible for monitoring medical devices in accordance with the Medical Devices Operator Ordinance. These are in contact with each other via the AGMP (Medical Devices Working Group) of the ZLG (Central Institute of the Federal States for Health Protection) (https://www.zlg.de). Which state authority is responsible for enforcing the Medical Devices Operator Ordinance varies from state to state (in Bavaria, for example, the State Office for Weights and Measures is responsible, in Baden-Württemberg the Verification and Certification Office). 

Medical practice in Germany is not subject to state supervision. The self-administration of the medical profession is the responsibility of the state medical associations. 

Within the framework of medical self-administration, laboratory diagnostics are currently mainly monitored by the Associations of Statutory Health Insurance Physicians. 

Within the framework of statutory health insurance physicians’ care, there is an agreement between the National Association of Statutory Health Insurance Physicians (KBV) and the National Association of Statutory Health Insurance Funds (GKV) on quality assurance measures for the provision of special examinations in laboratory medicine (Quality Assurance Agreement Special Laboratory, [4], [5] in accordance with Section 135 (2) SGB V, as Annex 3 of the Federal Coverage Agreement); in particular, this regulates the review of internal and external quality assurance measures in the outpatient care sector by the associations of statutory health insurance physicians of the chamber districts. In addition, providers of laboratory services are randomly inspected with regard to the Rili-BÄK. In 2022, 1,370 random sample and documentation checks were carried out. The respective laboratory commissions of the KVs are responsible. 

More specialized laboratory services (Chapter 32) are among the services requiring approval; accordingly, doctors who provide these services (e.g. gynecologists for hormone analysis) must provide separate proof of specific qualifications to the respective associations of statutory health insurance physicians or pass an examination. 

In addition to the Rili-BÄK, the G-BA’s general quality management guideline is also binding for medical laboratories within the framework of statutory health insurance; compliance with this guideline is monitored by the Medical Service. The G-BA has not yet issued a quality assurance guideline for medical laboratory tests.

Active, voluntary quality assurance of medical laboratories is mainly carried out through accreditation in accordance with the DIN EN ISO 15189 standard by the German Accreditation Body (DAkkS). In the actual sense, EU law provides for the accreditation of laboratories that carry out conformity assessment procedures – for example in the field of hygiene – while laboratory diagnostics as a diagnostic medical activity is not to be regarded as a conformity assessment. Nevertheless, accreditation in accordance with ISO 15189 is now very widespread and has been implemented practically across the board in the area of laboratories in private practice. If accreditation exists, laboratories are not inspected by the Associations of Statutory Health Insurance Physicians with regard to quality assurance. As of 2024, the DAkkS holds around 450 accreditation certificates for medical laboratories. Accreditation is a voluntary procedure, but is then carried out by the authorities. A statutory scale of fees applies. Expert assessors from the field of laboratory diagnostics work on a freelance basis for the accreditation.

The Genetic Diagnostics Act requires accreditation for paternity tests. For individual areas such as newborn screening, accreditation in accordance with ISO 15189 is also a billing requirement. Certification of laboratories in accordance with the general management standard ISO 9001 is not widespread; such certifications are explicitly not professionally oriented and therefore may not constitute a professional certification. 

The placing on the market of in-vitro diagnostics (devices, reagents, control materials, etc.) is regulated at EU level by the IVD Regulation (IVDR; Regulation (EU) 2017/746). This is differentiated according to four defined risk classes. For most of the products placed on the market, compliance with general performance and safety requirements under IVDR must be independently determined by notified bodies (IVD CE certification). The notified bodies are private-sector service providers that are subject to state supervision in the EU member states. The IVDR also addresses IVD articles that are manufactured by laboratories exclusively for their own use, but not the performance of testing procedures, which are to be regulated at EU member state level.

Certification as a doctor and medical licensing – as a prerequisite for specialist training – is carried out by the state (state examination offices, regional councils, etc.). With regard to the competence of doctors working in laboratory diagnostics, quality assurance is primarily based on the procedures for specialist recognition within the framework of medical self-administration. It is based on the further training regulations and the examination procedures of the state medical associations. The further training regulations of the state medical associations are based on the model further training regulations of the German Medical Association. The further training regulations are competence-based.

Continuing medical education (CME) in accordance with Section 95d SGB V is mandatory for doctors who work in the provision of care for people with statutory health insurance after obtaining specialist certification. General and specialist content is addressed. There is no periodic review of specialist competence by the medical associations in the sense of repeat examinations in Germany. 

## 8 IVD-industry

Five manufacturers worldwide offer comprehensive total solutions for highly automated large laboratories on the global market (Roche, Siemens, Abbott, Beckman, Ortho Clinical Diagnostics). Large Chinese manufacturers are also increasingly appearing internationally (e.g. Mindray). Comprehensive system solutions include the photometry and electrochemistry modules known as “clinical chemistry” as well as immunoassay modules. Comprehensive automation solutions for sample preparation and archiving are now also available from several providers in coherent, modular overall automation configurations (LAS, Laboratory Automation Systems). These configurations are freely scalable. Modules for hematology/cell counting and coagulation analysis can also be integrated. Complete solutions can either be provided by individual suppliers or configured modularly from devices from different manufacturers. The modules mentioned are also available as individual systems for smaller laboratories – particularly in clinics. A number of manufacturers offer primary stand-alone devices for more specialized analysis spectra and techniques (e.g. Diasorin, IDS, Werfen, Sysmex, Sebia, Tosoh; immunoassays, electrophoresis systems, HPLC systems); some of these systems can also be integrated into an LAS. In dedicated specialty analytics, there is a more complex supplier situation of small and medium-sized manufacturers (e.g. Chromsystems and Recipe in chromatography, Tecan in special immunoassays).

The production of in-vitro diagnostics also takes place to a significant extent in Germany. Large manufacturers (including Roche Diagnostics, Siemens, Abbott, Beckman Coulter) are active in this field, as are a large number of small and medium-sized companies. Within the EU, the IVD industry is most strongly represented in Germany.

On its website in January 2024, the German Diagnostics Industry Association (VDGH) stated that the total market volume of the diagnostics industry in Germany in 2022 was € 3.54 billion (compared to € 6.30 billion in 2021 due to the pandemic) [22]. Of this, 89.8% was accounted for by reagents and 10.2% by devices and services. For reagents, the size of the submarkets is as follows: infectiology 1.62 billion, immunochemistry 612 million, clinical chemistry 473 million, hematology 260 million, microbiology 170 million, genetic testing 36.5 million. 

In a press release dated March 27, 2024, the VDGH states that the industry generated € 4.1 billion in coronavirus diagnostics in 2021, € 1.29 billion in 2022 and € 110 million in 2023.

According to the VDGH [22], around 31,000 people are employed in the diagnostics industry in Germany. In 2020, the proportion of companies’ expenditure on research and development amounted to around 10% of turnover.

## 9 Universities and qualifications

In the licensing regulations for doctors, laboratory medicine is primarily addressed in the subjects of clinical chemistry and laboratory diagnostics, as well as in the subjects of microbiology and pathology. While chairs for microbiology and pathology have traditionally been represented at practically all medical faculties for a long time, this is not the case for clinical chemistry. Chairs have been established here in Germany mainly since the 1960s. The DGKL e.V.’s Research and Teaching Atlas of Universities in Laboratory Medicine 2022 provides an overview [23]. At faculties without their own chair, compulsory teaching (with lectures, seminars and practicals) is carried out by lecturers. At most universities, structurally integrated teaching of the individual sub-subjects of in-vitro diagnostics has not been realized, although such a concept certainly has potential. The approbation order subjects of laboratory diagnostics are examined in the written and oral parts of the state examination (medical examination), whereby the sub-questions cannot be explicitly assigned to the subjects.

Only a few specific technical-analytical degree courses in Germany have so far addressed medical analysis within bioanalytics to a certain extent. A relatively large – but not reliably quantifiable – number of non-physician academics work in laboratory diagnostics in Germany, on the basis of degree courses such as chemistry, food chemistry, biology, biochemistry and chemical engineering. The prerequisite for the performance and supervision of reserved activities (see above) is the demonstrable acquisition of the relevant knowledge for diagnostics. A state-recognized post-graduate qualification for analysts in medical diagnostics does not exist in Germany. The qualification “clinical chemist” is taught according to a defined curriculum of the German Society for Clinical Chemistry and Laboratory Medicine (DGKL) by authorized training institutions; however, it is not state-recognized. 

Training to become a medical technologist for laboratory diagnostics (MTL) takes place at vocational schools and not at universities. MTL schools are traditionally affiliated with clinics or are now run by private providers. Responsibility for these schools lies with the respective federal states. The training period is three years, with a now extensive practical component; a training allowance is paid. Academization of MTL training (corresponding to the basic academization of midwifery and obstetric nursing training, for example) has been under discussion for many years, but is not in sight. There are still very few opportunities for MTLs to gain specific higher qualifications at universities in Germany. As MTL schools are run by the authorities at state level, the nationwide number of currently operating schools or ongoing training courses is not available.

## 10 Laboratory diagnostic research

Specific laboratory diagnostics research takes place primarily at university departments [23], as well as in the diagnostics industry. Among other things, it relates to the translation of innovative analysis techniques in routine diagnostics – for example in mass spectrometry. The identification of new biomarkers is addressed at the interface between diagnostics and basic research. Clinical research related to laboratory diagnostic tests that are already technically established is addressed by many medical disciplines, often in collaboration between clinical study partners and laboratory diagnosticians. Collaboration between diagnostics manufacturers and clinics also plays an important role in laboratory diagnostics research. A significant part of laboratory diagnostics research currently addresses complex multi-parametric analysis techniques that involve comprehensive biochemical fields – often characterized as “omics techniques”. An important role for the application of artificial intelligence is foreseeable.

The number of tests that have been successfully translated from research into routine diagnostics over the last two decades cannot be stated exactly, but is relatively moderate compared to the number of existing procedures. These include natriuretic peptides in the diagnosis of heart failure, PCT and interleukin-6 in the diagnosis of sepsis, anti-CCP antibodies in rheumatology, AMH in reproductive medicine and calprotectin in the diagnosis of chronic inflammatory bowel disease. 

## 11 Associations in laboratory diagnostics

Among the associations active in the field of laboratory diagnostics, a distinction must be made between scientific societies on the one hand and professional associations on the other.

The scientific societies include the German Society for Clinical Chemistry and Laboratory Diagnostics (DGKL, https://www.dgkl.de), the German Society for Hygiene and Microbiology (DGHM, https://www.dghm.de) and the Society for Virology (GfV, https://www.g-f-v.org). Specialist societies that are also, but not exclusively, dedicated to laboratory diagnostics are the German Society for Transfusion Medicine and Immunohaematology (DGTI, https://www.dgti.de), the German Society for Pathology (DGP, https://www.pathologie-dgp.de), the German Society for Haematology and Oncology (DGHO, https://www.dgho.de) and the German Society for Immunology (DGI, https://dgfi.org). The umbrella organization of the specialist societies in Germany is the Association of the Scientific Medical Societies in Germany (AWMF, https://www.awmf.org); there are no personal memberships here. A functional unit of the AWMF is the Ad-hoc Commission on In-vitro Diagnostics, which is dedicated in particular to interdisciplinary networking. 

The European Federation of Laboratory Medicine (EFLM, https://www.eflm.eu) is the umbrella organization of the European professional societies for clinical chemistry and laboratory diagnostics, while the International Federation of Clinical Chemistry (IFCC, https://ifcc.org) is the global umbrella organization. Here, too, there are only corporate memberships. The leading global professional association for laboratory diagnostics is the Association for Diagnostics and Laboratory Medicine (ADLM, https://www.myadlm.org), which is primarily active in the USA.

In contrast to the scientific medical associations, the professional associations are usually not non-profit organizations according to their statutes. In the field of laboratory diagnostics, the following should be mentioned in particular: the Dachverband der Technologen/-innen und Analytiker/-innen in der Medizin Deutschland (dvta, https://www.dvta.de), the Berufsverband Deutscher Laborärzte (BDL, https://www.bdlev.de), the Berufsvereinigung der Naturwissenschaftler in der Labordiagnostik (BNDL, https://www.bnld.de), the Professional Association of Physicians for Microbiology, Virology and Infection Epidemiology (BÄMi, https://www.baemi.de), the Association of Independent Laboratories (AULA, https://aula-labore.de), Ärztliches Qualitätslabor (ÄQL, https://www.aeql.de).

The association ALM e.V. (Akkreditierte Labore in der Medizin, https://www.alm-ev.de), an association of accredited medical laboratories in Germany, has the most public visibility among the professional associations in the field of laboratory diagnostics. According to its own information, the association represents around 900 medical specialists and around 500 scientists, and around 25,000 qualified employees. ALM lists 20 corporate members with over 200 laboratory locations on its website. All major laboratory associations and individual owner-managed laboratories are represented. The largest members include the providers Sonic Healthcare (Australian stock corporation, 37,000 employees worldwide, 7,500 in Germany, over 50 locations), Synlab (listed stock corporation based in Germany, internationally active, over 200 medical specialists, over 30 locations in Germany), Limbach Group (more than 300 medical specialists, more than 5,000 employees, more than 30 laboratories). ALM represents laboratory diagnostics in the SpiFA, the umbrella organization of professional medical associations.

## 12 Discussion and outlook

Laboratory diagnostic tests are by far the most frequent technical measure in medicine; the structures of laboratory diagnostics can be described as systemically relevant for medicine in Germany, as they affect practically all medical institutions. Laboratory diagnostics – with its non-curative, secondary medical subspecialties – represents a central, highly effective and efficient pillar of evidence-based medicine. 

From publicly available sources of information, a relatively meaningful picture of this branch of medicine in Germany can be drawn. However, it should be noted that the structures of laboratory diagnostic service provision in the various sectors are quite heterogeneous – they range from near-patient immediate diagnostics to highly specialized diagnostics, for example in oncological haematology, to highly centralized, large-scale laboratories operated by financial investors. Accordingly, healthcare research in laboratory diagnostics in Germany shows a system-inherent lack of certainty – e.g. with regard to the actual total number of all analyses carried out annually, or the extent of institutional quality assurance by authorities.

Overall, the volume of laboratory diagnostics services in Germany is very high. There are no obvious indications of relevant gaps in care or quality problems, although there is no systematically collected data on this from the perspective of curative physicians. 

The number of laboratory physicians has risen by around 21% over the past 13 years, which is lower than the percentage increase in the number of specialists overall (Table 1 (Tab. 1)). 

The costs spent on laboratory diagnostics in healthcare rose by 55% between 2012 and 2020, which is relatively lower than the 64% increase in costs for the entire healthcare system in Germany (Table 2 (Tab. 2)). This trend of cost growth is undoubtedly a systemic threat to healthcare in Germany and this must also be addressed in laboratory diagnostics in order to prevent future rationing of healthcare services. 

An international comparison of the scope and quality of laboratory diagnostic care is difficult due to a lack of comparable publications. A comprehensive report is only available for the USA [24], although this dates from 2008. 

A fundamental problem of laboratory diagnostic care in Germany is the increasingly relevant shortage of specialists. This can partly be explained by demographics. Germany-wide statistical data on MTL schools and their situation is not available due to the federal structures of the school system. The new regulation of professional training in the analytical-technical field by the 2021 Act on Professions in Medical Technology (MT-Berufe-Gesetz, MTBG) poses considerable challenges for training institutions; it is not yet possible to predict with certainty how this will affect the number of graduates. The MTBG still stipulates that the essential, analytically and technically demanding activities in the medical laboratory may only be carried out by medical laboratory technologists; however, the opening with regard to academic-analytical qualifications is becoming increasingly relevant.

An increasing partial substitution of MTL by semi-skilled personnel or personnel from other medical professions (especially medical assistants (MFA) and biotechnologists) under supervision is to be expected. This undoubtedly represents a growing challenge for skills management in medical laboratories. 

In addition to an increasing shortage situation in the MTL sector, increasing staff shortages are also to be expected in the medical laboratory sector. Although the demand planning of the Associations of Statutory Health Insurance Physicians currently still shows an oversupply in all planning areas, an increasingly tense personnel situation is to be expected in the coming decade due to the given age distribution of those working in the field. It is therefore particularly important to introduce young doctors to the field of laboratory diagnostics as a non-curative area of medicine – ultimately in order to maintain the traditionally dedicated medical character of laboratory diagnostics in Germany.

Medicine in Germany is characterized by a continuous increase in pressure to provide care across all disciplines; this is mainly due to a demographically induced increase in morbidity and a simultaneous, also demographically induced decrease in the number of service providers. At present, laboratory diagnostics is obviously better able to withstand this pressure than other disciplines. There is no perceptible public discussion about performance deficits in laboratory diagnostics – in stark contrast to curative specialist care in many areas or in the area of acute care in emergency outpatient clinics. The structural and process quality of laboratory diagnostics in Germany can currently be rated as very good overall. Increasingly efficient automation of laboratory analysis and consolidation of laboratories on the basis of increasingly optimized sample transport logistics can currently prevent a shortage of laboratory diagnostics in Germany. Nevertheless, it is imaginable that smaller laboratory locations will have to be abandoned due to a shortage of MTL for reserved activities. 

Overall, laboratory diagnostics in Germany is increasingly dominated by large, supra-regional or international laboratory networks, ultimately of a commercial nature – both in the outpatient and inpatient sectors. In line with general economic market mechanisms, further market consolidation can be expected here, as larger units are generally able to realize more pronounced synergy effects; the increasing shortage of specialists could also have an impact on such a development. In fact, the concentration process of medical laboratories has also progressed in recent years.

The role of international financial investors in German medicine in general is being perceived increasingly critically – at least by the medical public. These investors now play a major role in laboratory diagnostics – far more so than in other areas of medicine. On the basis of this capital commitment, profits are to be drawn from the solidarity system of German medicine. Investors emphasize that their involvement provides the healthcare system with capital for patient care that can no longer be provided by the system. There is a consensus that medical activity in diagnostics – as in the curative field – must not be compromised by commercial interests and considerations, and that the medical character of laboratory diagnostics in German medicine must be preserved. The protection of the independence of medical decision-making in the laboratory (especially with regard to indications) and the prevention of anti-competitive provider dominance are important objectives of legislative projects announced in 2024.

From the commercial perspective of investors, the question can also be raised as to whether laboratory diagnostics can be assumed to be a growth area in Germany. This is conceivable in view of a demographic-related increase in morbidity; in particular, the increase in the prevalence of diabetes mellitus and its secondary diseases must be taken into account. On the other hand, approaches to “utilization management” in laboratory diagnostics with the aim of more efficient use of tests have so far only been pursued marginally. The extent to which areas of overuse and inappropriate use of laboratory services can be assumed in Germany is not yet the subject of published health services research.

To a certain extent, an increase in performance in laboratory diagnostics can be expected as a result of new techniques. This applies, for example, to nucleic acid-based techniques in oncology. Companion diagnostics methods are used to enable the personalized use of drugs that target specific tumour mutations. The analysis of circulating nucleic acid chains is increasingly being used for therapy monitoring, but is also of growing interest with regard to potential malignant tumor screening. In general, with regard to innovative nucleic acid-based laboratory diagnostic procedures, it can be observed that the primarily sharp professional boundary between the disciplines of clinical chemistry/laboratory medicine and pathology is disappearing. 

Therapeutic drug monitoring (TDM) can be assumed to be a growth area – the concept of individually adjusting the dosage of (low and high molecular weight) drugs based on drug level measurements. This area has been little addressed by the diagnostics industry for decades. On the part of the pharmaceutical manufacturers, a fundamentally conservative or even negative attitude towards this area of diagnostics can be observed. However, in some areas – e.g. antibiotic treatment of life-threatening infections – there is now a clear clinical need. Mass spectrometry is clearly emerging as a key technology for TDM. Increasing automation will probably significantly increase translation into standard laboratories in the coming years. A mass spectrometric “omics” technique is now an industrialized routine procedure in bacteriology; for decades, various other “omics” approaches have been developed and investigated to see whether they can make a useful contribution to clinical laboratory diagnostics in the future. “Omics” stands for analytical methods that record a large number of analytes, often from different chemical classes, as qualitative or quantitative samples (protomics, metabolomics, lipidomics, etc.). 

Laboratory diagnostic services are usually ordered by doctors according to evidence-based indications. Analysis commissioned directly by “consumers” has a potentially increasing scope (DTC, direct-to-customer). This includes nutrition-related tests (e.g. trace element status, vitamin status), “complementary medicine tests” (often ordered by alternative practitioners), but also genetic analyses. This area of analysis is highly questionable from a medical point of view, especially as results that deviate from normal collectives can represent a relevant burden for those affected without actually being relevant to their health. The same applies in principle to so-called individual health services (IGeL services); these are examinations offered by doctors, although in specific cases they are not included in the scope of services provided by the statutory health insurance funds – due to a lack of evidence-based evidence (e.g. vitamin D screening, gynaecological tumor-associated proteins in healthy individuals, extensive vitamin and trace element analyses). In this area, the indication quality of the requirements is often very questionable. Also problematic from a medical point of view is the extension of the range of services offered by pharmacies to include laboratory diagnostics, which is sometimes propagated politically. At the very least, the indication and assessment of laboratory tests should be seen as a reserved medical activity.

In the area of hospital laboratories, the upcoming hospital reform will probably bring about some changes; it is to be expected that the number of hospitals in Germany will decrease. This is likely to affect small institutions, only a small proportion of which have their own laboratory infrastructure. Overall, only well under 20% of clinics now have their own laboratory infrastructure. Immediate patient-oriented diagnostics (POCT) is accordingly combined to a large extent with external laboratory cooperations – with other clinics or laboratory practices. As the availability of MTL in clinics presumably continues to decrease and technical equipment solutions continue to improve, the importance of POCT – over and above classic blood gas and glucose analysis – will presumably increase. This trend will increase the technical demands on nursing staff. For a large part of extended routine analysis, sample transportation to laboratories in private practice is tolerable, albeit not ideal, with a time horizon of approx. 12 hours.

If a hospital with an emergency department is unable to measure TSH, for example, to detect severe hypothyroidism, this can have negative consequences in individual cases. A relevant time delay due to sample outsourcing is particularly critical in the area of infection diagnostics based on blood cultures. To a certain extent, it can be expected that sample transportation using drones will make a useful contribution to logistics and the networking of hospital laboratories in conurbations in the future. 

As of the end of 2024, the German laboratory medical profession considers changes and redistributions in the EBM from 2025 onwards to be very critical, or in some cases threatening to their existence as a result of an expected significant deterioration in the fee situation. The upcoming introduction of electronic patient files in the context of the MIO standard (Medical Informatics Objects) for the exchange of laboratory results also represents a current challenge.

Overall, the structures of medical laboratory diagnostics in Germany – which is very heterogeneous compared to other medical disciplines – have been subject to a high level of dynamism in recent years, which is likely to continue in the coming years.

## Notes

### Use of language

For better readability, the generic masculine is used in some parts of this work. Unless otherwise indicated, the personal designations used refer to all genders.

### Competing interests

The authors declare that they have no competing interests.

## Figures and Tables

**Table 1:**
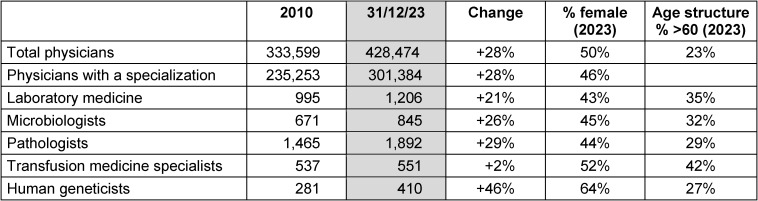
Number of Employed Physicians. Excerpt from the Registers of the German Medical Association [1], as of the end of 2010 and 2023

**Table 2:**
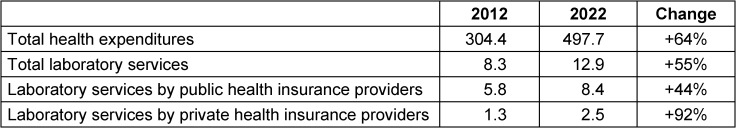
Health expenditure accounting in billion euros according to the Federal Statistical Office, Destatis [16] (query as of 20/09/2024)
